# The Complete Exosome Workflow Solution: From Isolation to Characterization of RNA Cargo

**DOI:** 10.1155/2013/253957

**Published:** 2013-09-25

**Authors:** Jeoffrey Schageman, Emily Zeringer, Mu Li, Tim Barta, Kristi Lea, Jian Gu, Susan Magdaleno, Robert Setterquist, Alexander V. Vlassov

**Affiliations:** Life Technologies, Austin, TX 78744, USA

## Abstract

Exosomes are small (30–150 nm) vesicles containing unique RNA and protein cargo, secreted by all cell types in culture. They are also found in abundance in body fluids including blood, saliva, and urine. At the moment, the mechanism of exosome formation, the makeup of the cargo, biological pathways, and resulting functions are incompletely understood. One of their most intriguing roles is intercellular communication—exosomes function as the messengers, delivering various effector or signaling macromolecules between specific cells. There is an exponentially growing need to dissect structure and the function of exosomes and utilize them for development of minimally invasive diagnostics and therapeutics. Critical to further our understanding of exosomes is the development of reagents, tools, and protocols for their isolation, characterization, and analysis of their RNA and protein contents. Here we describe a complete exosome workflow solution, starting from fast and efficient extraction of exosomes from cell culture media and serum to isolation of RNA followed by characterization of exosomal RNA content using qRT-PCR and next-generation sequencing techniques. Effectiveness of this workflow is exemplified by analysis of the RNA content of exosomes derived from HeLa cell culture media and human serum, using Ion Torrent PGM as a sequencing platform.

## 1. Introduction

Exosomes are a type of vesicle, 30–150 nm in size, that have received increased attention over the past few years [1–7]. Exosomes are secreted by all cell types in culture and also found naturally in blood, urine, cerebrospinal fluid, breast milk, saliva, ascitic fluid, and amniotic fluid in very high numbers [7, 8]. Depending on the cell of origin, many different functions have been attributed to exosomes such as morphogen transporters in the creation of polarity during development and differentiation [4], role in programmed cell death, angiogenesis, inflammation, coagulation [9], and migration of *Dictyostelium* cells by the secretion of chemoattractant signals [10]. Valadi et al. [11] demonstrated that MC/9 and HMC-1 mast cells secrete exosomes that contain mRNA from approximately 1300 genes and small RNAs, including 121 unique microRNAs. The transfer of exosomes to a donor cell showed that at least some mRNAs were full length, as they were translated in the recipient cell. Glioblastoma cells also secrete exosomes and microvesicles containing mRNA, miRNA, and angiogenic proteins [12]. When taken up by host human brain microvascular endothelial cells, mRNA molecules were translated and tubule formation by the target endothelial cells was stimulated. The spread of oncogenes by exosomes and microvesicles secreted by tumor cells has been reported [13]. Exosomes also play a crucial role in disseminating pathogens such as prions and viruses from one cell to another [14–17]. Interest towards exosomes, from their function in the body to more practical applications, such as the use in diagnostics (based on analysis of their RNA and protein content) and therapeutics development, has grown exponentially in the last five years [18–20]. 

Despite a rapidly growing number of research studies, there is still rather limited and superficial information available regarding RNA content of exosomes [21]. Several reports have been published to date, utilizing sequencing technologies to characterize the RNA content of exosomes derived from THP1 and HUVEC cells [22], DC cells [23], hES-MSC cells [24], placenta [25], and breast milk [26]. Except for Nolte-'t Hoen et al. [23], who characterized a wide population of small RNA residing within the vesicles, all studies solely focus on analysis of the miRNA content. Significantly less information is available regarding other RNA classes and other types of body fluids, such as blood. Moreover, certain reports contain a number of contradictions. Several papers, for example, indicate that the RNA “cargo” of exosomes is substantially different from the parental cell content [12, 27, 28]. This runs counter to several other authors working with cancer cells, who have noted that the miRNA content for their originating cancer cells is similar to that found in circulating exosomes, opening up the possibility that exosomes could be used as unique diagnostic markers [29, 30]. The complicating factor between these studies is a lack of standardized techniques, protocols, and workflows for isolation of exosomes and downstream analysis of their constituents. Currently the most popular approach for isolation of exosomes is based on ultracentrifugation [31], which allows the researcher to obtain highly pure exosomes; however, it is a very lengthy, difficult, and unreliable process. To address the urgent need to have a simple solution for isolation of exosomes, several reagents were developed by biotechnology companies in the last two years. System Biosciences released a proprietary reagent named ExoQuick that can be added to serum, conditioned cell media, or urine and is claimed to precipitate the exosomes [32]. HansaBioMed is offering an array of products called ExoTest kits—featuring anti-CD63, -CD81, or -CD9 antibodies immobilized on 96-well plates—for exosome capturing and characterization [33]. Bioo Scientific launched the ExoMir kit that essentially removes all cells, platelets, and cellular debris on one microfilter and captures all vesicles bigger than 30 nm on a second microfilter using positive pressure to drive the fluid [34]. One should keep in mind that, depending on the isolation procedure, exosome preparations are contaminated to a various extent with other microvesicles or RNA-protein complexes [35], dramatically affecting the outcome of downstream analysis.

Here we describe a complete exosome workflow solution, starting from fast and efficient extraction of exosomes from cell culture media and serum to robust isolation of RNA and characterization of exosomal RNA content using qRT-PCR and next-generation sequencing techniques. Effectiveness of this workflow is demonstrated by analysis of the RNA content of exosomes derived from HeLa cells and blood serum, using Ion Torrent PGM as a sequencing platform. This straightforward standardized workflow can be utilized for analysis of the exosomal RNA cargo, shedding some light on the mechanisms of formation, preferential loading, and functions of these fascinating vesicles. In a similar fashion, disease-specific RNA signatures residing within the exosomes, can be uncovered and potentially used for development of minimally invasive or noninvasive diagnostics, providing the possibility of someday being able to analyze RNA “content” of organs and tissues without biopsy. 

## 2. Materials and Methods

### 2.1. Materials

Total exosome isolation (from serum) reagent (Invitrogen), Total exosome isolation (from cell culture media) reagent (Invitrogen), Total exosome RNA and protein isolation kit (Invitrogen), blood serum from two donors, cell culture media from HeLa cells, 10x PBS, nuclease-free water (Ambion), 100% ethanol, nonoptical adhesive covers (Applied Biosystems), optical adhesive covers (Applied Biosystems), 384-well PCR standard plates (Applied Biosystems), 96-well PCR standard plates (Applied Biosystems), universal PCR master mix II (Applied Biosystems), human TaqMan miRNA assays, Veriti 96-well thermocyclers (Applied Biosystems), 7900HT Instrument, SW v2.3, TaqMan microRNA reverse transcription kit (Applied Biosystems), 1000 reactions, and Ion Total RNA-Seq kit v2 (Life Technologies) were utilized.

### 2.2. Extraction of Exosomes from Serum and Cell Media Using Total Exosome Isolation Reagents

#### 2.2.1. Cell Culture Media

Fresh cell media was harvested from HeLa cells, grown in T175 flasks. Initially, the cells were grown in media containing 10% FBS (to ~90% cell density), then washed twice with PBS and grown for the remaining 12 h in 10% exosome-depleted FBS. The cell media samples were then centrifuged at 2,000 g for 30 min to remove cell debris. The supernatant containing the cell-free cell media was transferred to a fresh container and held on ice until use. Next, each sample was combined with 1/2 volume of Total exosome isolation (from cell media) reagent and mixed well by vortexing or pipetting up and down until a homogenous solution was formed. Typical cell media volume utilized was 1 mL; however, the range of 100 *μ*L–50 mL was used depending on the downstream application. The samples were incubated at 4°C overnight and then centrifuged at 4°C at 10,000 g for 1 h. The supernatant was aspirated and discarded, and the exosome pellet was resuspended in PBS buffer and then stored at 4°C short term (1–7 days) or −20°C long term. 

#### 2.2.2. Human Blood Serum

Frozen serum samples were thawed in a water bath at room temperature until samples were completely liquid and then centrifuged at 2,000 g for 30 min to remove any cellular debris. The supernatant containing the cell-free serum was transferred to a fresh container and briefly held on ice until use. Next, each serum sample was combined with 1/5th volume of Total exosome isolation (from serum) reagent and then mixed well by vortexing or pipetting up and down until a homogenous solution was formed. Typical serum volume utilized was 100 *μ*L; however, the range of 50 *μ*L–5 mL was used depending on the downstream application. The samples were incubated at 4°C for 30 min and then centrifuged at room temperature at 10,000 g for 10 min. The supernatant was aspirated and discarded, and the exosome pellet was resuspended in PBS buffer and then stored at 4°C short term (1–7 days) or −20°C for long term. 

### 2.3. Sizing and Quantification of Exosomes with Nanosight LM10 Instrument

Exosomes purified from cell media and blood serum were diluted with PBS buffer (10–5000x in order to have the nanovesicle concentration in the working range for the Nanosight LM10, 2 × 10^8^–8 × 10^8^) and then quantified and sized using the Nanosight LM10 instrument (Nanosight, UK), following the manufacturer's protocol. The LM10 uses a laser light source to illuminate nanoscale particles (10–1000 nm) which are seen as individual pointscatters moving under Brownian motion. The paths of the point scatters, or particles, are calculated over time to determine their velocity which can be used to calculate their size independent of density. The image analysis NTA software compiles this information and allows the user to automatically track the size distribution and number of the nanoparticles. 

### 2.4. Western Blot Analysis

Exosome samples isolated from cell media or blood serum (typically equivalent of 50 *μ*L cell media and 5 *μ*L serum) were mixed with 2x nonreducing Tris-glycine SDS sample buffer (Novex) for CD63, and 2X reducing buffer for CD9, then heated at 75°C for 5 min and loaded onto a 1.5 mm × 15 well 4–20% Tris-Glycine gel (Novex). Benchmark prestained protein ladder (Invitrogen) was added to one well as a control to monitor the molecular weight of the protein samples. The gel was run under denaturing conditions at 150 V for 1.5 h and then transferred to a membrane using the iBlot instrument (Life Technologies). After transfer, the membranes were processed on the BenchPro 4100 (Life Technologies) with CD63 or CD9 antibody (Abcam) diluted 100 *μ*g into 20 mL. The WesternBreeze Chemiluminescence kit was utilized on the next step; membranes were exposed to X-ray film for 1–10 min and the film was analyzed.

### 2.5. RNA Recovery Using the Total Exosome RNA and Protein Isolation Kit

The Total exosome RNA and protein isolation kit (Invitrogen) was utilized for recovery of RNA from the exosome samples obtained with the reagent and ultracentrifugation protocol and parental samples for each sample type, HeLa cell pellets (1 × 10^6^ cells) and cell-free serum. 200 *μ*L of each sample (brought up to volume with PBS if necessary) was combined with 205 *μ*L of 2x denaturing solution, vortexed to lyse, and then incubated on ice for 5 min. After incubation, 410 *μ*L of acid-phenol : chloroform was added to the mixture and vortexed for 30–60 sec to mix. Samples were then centrifuged for 5 min at 10,000 g at room temperature to separate the mixture into aqueous and organic phases. Once centrifugation was complete, the aqueous (upper) phase was carefully removed without disturbing the lower phase or the interphase and transferred to a fresh tube. 

1.25 volume of 100% EtOH was added to the aqueous phase for each sample and then vortexed to mix. 700 *μ*L of volume was placed onto spin column in a collection tube and then spun at 10,000 g for 15 sec to move the sample through the filter cartridge. Samples were then washed once with 700 *μ*L wash solution 1 and 2x with 500 *μ*L wash solution 2/3 (centrifuged at 10,000 g for 15 sec for each wash). After washing, filter was dried by spinning for an additional 1 min at 10,000 g. The filter cartridge was transferred into a fresh collection tube and 50 *μ*L of preheated (95°C) nuclease-free water was applied to the center of the filter. Samples were centrifuged for 30 sec at 10,000 g to recover the RNA, and then a second 50 *μ*L volume of preheated (95°C) nuclease-free water was applied to the center of the filter and centrifuged for 30 sec at 10,000 g. After the second spin, the eluate containing the RNA was collected and stored at −20°C. For cell pellet RNA, a DNase treatment was performed using the DNase-free Kit (Ambion) to remove any contaminating DNA; DNase treatment was not performed on exosome samples as they had a much smaller input. After treatment, each sample was diluted to 2 ng/*μ*L and 1 *μ*L analyzed on the Agilent 2100 Bioanalyzer using the Agilent RNA 6000 Pico Kit (Series II) to determine the mass of RNA going into downstream analysis. 

### 2.6. Reverse Transcription and Quantitative Real-Time PCR (qRT-PCR) Analysis of the RNA Sequences Isolated from the Exosomes

Reverse Transcription (RT) Master Mix was prepared for each sample using the TaqMan MicroRNA Reverse Transcription Kit reagents and protocol (Applied Biosystems) with gene specific RT primers for five miRNA targets (miR16, miR24, miR26a, miR451, and let7e). Ten *μ*L of the RT master mix was added to corresponding wells in a 96-well plate, and 5 *μ*L of each sample was added to the master mix. Plates were covered with adhesive (nonoptical) cover and spun down to remove air bubbles and then placed into a 9700 thermocycler and incubated as follows: 4°C for 5 min, 16°C for 30 min, 42°C for 30 min, and 85°C for 5 min. Reactions were kept at 4°C until use.

qPCR master mixes were prepared for each of five microRNAs by combining 5 *μ*L of AB Universal PCR Master Mix II, 2.5 *μ*L of nuclease-free water, and 0.5 *μ*L of the 20x TaqMan assay. After mixing, 8 *μ*L of each master mix was placed into wells in a 384-well plate (enough for triplicate reactions for each isolation replicate). Two *μ*L of each RT reaction was added in triplicate to the master mix of each target and the plates were sealed with an optical adhesive cover. Plates were spun down to remove air bubbles and then placed into a 7900HT instrument and run using the following thermocycler protocol 95°C for 10 min + (95°C for 15 s; 60°C for 60 s) for 40 cycles. Once the run was complete, automatic Ct analysis was performed with SDS v2.3 software, and average and standard deviations were calculated for each set of isolations and qPCR reactions for each target.

### 2.7. Preparation of the Small RNA Libraries and Sequencing Exosomal RNA

Small RNA libraries were prepared using the Ion Total RNA-Seq Kit v2 (Life Technologies) protocol and materials. However, a number of modifications were introduced into the RNA-Seq protocol in order to accommodate the specific nature of the exosome samples: (1) relatively low amount of RNA and (2) majority of the RNA cargo being <200 nt in size. For library construction, the RNA sample was dried down to 3 *μ*L and then combined with the hybridization reagents and incubated at 65°C for 10 min and 16°C for 5 min. Ligation reagents were then added and the samples were incubated at 16°C for 16 h (overnight). After ligation, reverse transcription was performed: RT master mix was added to the samples, tubes were incubated at 70°C for 10 min, samples were snap-cooled on ice, the RT enzyme was added, and the samples were incubated at 42°C for 30 min. cDNA from the RT reaction was purified using the kit's clean-up module containing MagMAX Beads (5 *μ*L per well of a 96 well plate) and eluted in 12 *μ*L of nuclease-free water. Six *μ*L of the purified cDNA was combined with PCR primers and Platinum PCR SuperMix High Fidelity reaction mix was then placed in a thermocycler and amplified using the following protocol: 94°C for 2 min (94°C for 30 s, 50°C for 30 s, and 68°C for 30 s) 2 cycles; (94°C for 30 s, 62°C for 30 s, and 68°C for 30 s) 16 cycles; 68°C for 5 min. Once protocol was complete, reactions were stored on ice until purification. The amplified DNA (final library) for each sample was purified using the kit's clean-up module containing MagMAX Beads (5 *μ*L per well of a 96-well plate) and eluted in 10 *μ*L of nuclease-free water. Final libraries were stored on ice for the short term and at −20°C for long term. To assess the yield and size distribution, 1 *μ*L of the library was run on an Agilent DNA High Sensitivity chip (Agilent). The molar concentration of the library was determined with the Agilent 2100 Bioanalyzer Instrument Expert software and used to dilute libraries to correct concentration for sequencing. Sequencing was performed for each sample on the Ion Torrent PGM instrument using 318 chips (11,000,000 wells per chip) and the protocol listed in the Total exosome RNA and protein isolation kit (Invitrogen) with 160 flows (40 cycles). 

### 2.8. Sequence Data Acquisition and Preprocessing

Upon completion of each PGM sequencing run, the Torrent Suite software (http://ioncommunity.lifetechnologies.com/docs/DOCS-7189/) performed base calling from raw signals. Reads from polyclonal beads and low quality reads were filtered out, and 3′ adapter sequences were trimmed before the sequence was output into a FASTQ formatted file for analysis (http://en.wikipedia.org/wiki/FASTQ_format/). Contained within this file are the sequences (reads) which correspond to a single bead location on the Ion 318 chip and per base PHRED scaled [36] (http://en.wikipedia.org/wiki/Phred_quality_score/, http://ioncommunity.lifetechnologies.com/docs/DOC-2306/) quality values. 

With the data in the FASTQ format, it was entered into the exosome-seq mapping pipeline for final analysis (see Figure 4). While the Torrent Suite trims 3′ adapter and filters low quality reads quite effectively, some residual suboptimal sequences occasionally remain. For this reason, the first step in the pipeline includes 3′ quality and adapter trimming using the *FASTX-Toolkit* (http://hannonlab.cshl.edu/fastx_toolkit/) and *Cutadapt* [37] programs, respectivel. Specifically, the *FASTX-Toolkit* subprogram *fastq_quality_trimmer *scans each read and its respective quality values from the 5′ to the 3′ end of the reads and trims bases below a PHRED quality value of 17. If the read length falls below 17 bases in length, it is removed from further processing. This is to reduce the number of ambiguously mapped reads which become more frequent as reads get shorter. The remaining reads are scanned for P1 adapter sequences at the 3′ end using *cutadapt*. Again, if the read length falls below 17 bases after adapter trimming, it is removed from analysis. 

These preprocessing steps are critical since low quality sequence and 3′ adapter sequences can be a source of misalignment (noise) or preclude the read from mapping at all which causes problems when trying to map reads to the locations in the genome from which they are transcribed.

### 2.9. Data Analysis: Quality Control and Assessment

After preprocessing each read of the FASTQ file was examined based on global quality metrics using fastqc http://www.bioinformatics.bbsrc.ac.uk/projects/fastqc/. The resulting graphs and statistics were based on quality values, nucleotide composition, sequence length, and most highly represented sequences (k-mers) for each library. This analysis provided a high-level evaluation of sequence quality and potential biases which could be present in a given RNA-Seq sample. If irregularities are observed, such as quality value distributions shifted lower, this may be an indication that a problem has occurred upstream from the analysis pipeline. 

### 2.10. Data Analysis: Iterative Mapping

As mentioned earlier, the goal of the sequencing and analysis is to determine measurable global expression profiles using multiple reference datasets. The alignment steps in this workflow utilize the *SHRiMP2* [38] aligner to map the FASTQ sequences to miRBase precursors. The *SHRiMP2* aligner was chosen as it has been tested and optimized specifically for mapping miRNA sequences using special parameters provided by the authors and can be implemented to align longer sequences with high sensitivity. The alignment algorithm used attempts to optimally align each read sequence to a reference containing miRNA hairpins (miRBase build 18.0), tRNA, and ribosomal RNAs and then report each alignment in a sequence alignment/map (SAM) (http://samtools.sourceforge.net/SAM1.pdf) formatted file. This file format is the current standard alignment format, and numerous downstream analysis tools have been written to use it for easy parsing and counting of reads to individual transcripts. 

Previously published reports [11, 39] have shown that mRNAs are also found in isolated exosome fractions and may be effectively quantitated through sequencing. To examine mRNA levels in exosomes compared to parental cell samples, a secondary mapping step was completed using unmapped reads from the previous miRNA mapping step as input for alignment to RefSeq (build Jan. 2012) transcripts, the majority of which are protein coding mRNAs. 

Finally, in order to comprehensively account for additional known RNA species which could be present in exosome fractions, the reads that did not map in the RefSeq step were taken and mapped to the noncoding RNA database Noncode (v2.0) [40]. This step allowed for the quantitation of piRNA, scaRNA, and snoRNAs not previously annotated by RefSeq.

To determine read counts matching each reference RNA at each step, a simple Perl script was written to scan each of the three SAM files and tally mapped read counts per transcript and then report them in tabular format. Once complete, these count tables were used to compare enrichment of particular transcript across sample types.

## 3. Results and Discussion 

### 3.1. Isolation and Initial Characterization of Exosomes from Cell Media and Serum

The original approach for isolation of exosomes, still widely used, is based on ultracentrifugation in combination with sucrose density gradients or sucrose cushions to float the relatively low-density exosomes away from other vesicles and particles [31]. These protocols range in time from 8 to 30 h and require an ultracentrifuge and extensive training to ensure successful isolation of exosomes. In addition, only six samples can be processed at a time, and the sample volume needed is quite large (typically 10–30 mL). As for a way to enable simple and fast exosome isolation, we developed two Total exosome isolation reagents. These reagents allow straightforward and reliable recovery of fully intact exosomes from cell culture media and blood serum samples, in a wide volume range, and are suitable for high throughput applications. By tying up water molecules, the reagents force less-soluble components such as nanovesicles out of solution. To isolate exosomes, the reagent is added to a biological sample, and the mixture is incubated at 4°C, followed by precipitation through the standard centrifugation at 10,000 g. The pellet containing the exosomes is then resuspended in PBS or similar buffer and the exosomes are ready for the end-point analysis or biological studies on their pathways and functions. 

**Figure 1:**
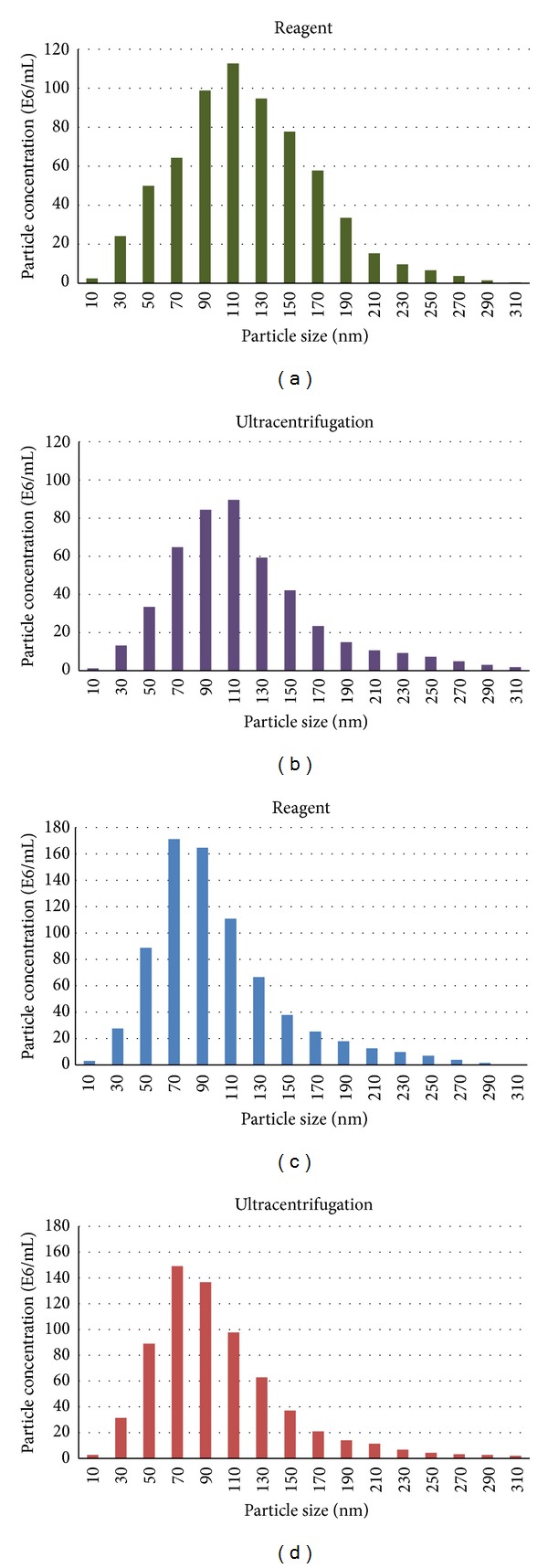
Both ultracentrifugation protocol and Total exosome isolation reagent enable recovery of very clean population of exosomes from cell media and serum. ((a) and (b)) Analysis of exosomes recovered from HeLa cell media using the Total exosome isolation reagent (from cell culture media) and ultracentrifugation protocol by Nanosight LM10 instrument. ((c) and (d)) Analysis of exosomes recovered from serum using the Total exosome isolation reagent (from serum) and ultracentrifugation protocol by Nanosight LM10 instrument.

We isolated exosomes from HeLa cell culture media (chosen as a model system as its use is ubiquitous in the laboratory and since a general understanding of this system had been achieved) and blood serum samples (derived from healthy human donors) using Total exosome isolation reagents as well as the ultracentrifugation procedure [31], for comparison. Sizing and quantification of exosomes were performed with the NanoSight LM10 instrument and results are shown in Figures 1(a) and 1(b) (cell media) and Figures 1(c) and 1(d) (serum). All nanoparticles recovered with both protocols were smaller than 300 nm, most of them being in the typical exosome size range of 30–150 nm. 

Both the reagent and ultracentrifugation procedures allowed recovery of a significant number of nanovesicles: HeLa cells were grown to ~2 × 10^7^ cells per T175 flask, in 30 mL cell culture media, in the presence of exosome-depleted FBS. From 1 mL of this cell medium, ~4–8 × 10^9^ exosomes were isolated with the reagent and ~2–4 × 10^9^ with ultracentrifugation. For the reagent protocol, 100 *μ*L−50 mL cell media volume inputs was tested, and exosome recovery was linear in this range. The process is scalable up or down depending on the needs for downstream analysis—for example, for sequencing applications, larger amounts of RNA are required, compared to qRT-PCR, and thus larger scale of exosome isolation is required, from 10–50 mL cell media.

From 100 *μ*L serum, ~1.5–3 × 10^11^ exosomes were recovered with the reagent and ~1.5–5 × 10^10^ with ultracentrifugation. For the reagent protocol, 50 *μ*L−5 mL serum volume inputs was tested, and exosome recovery was linear in this range. For sequencing applications, larger amounts of RNA are required, compared to qRT-PCR, and thus larger scale of exosome isolation is required, from 1–5 mL serum. 

**Figure 2:**
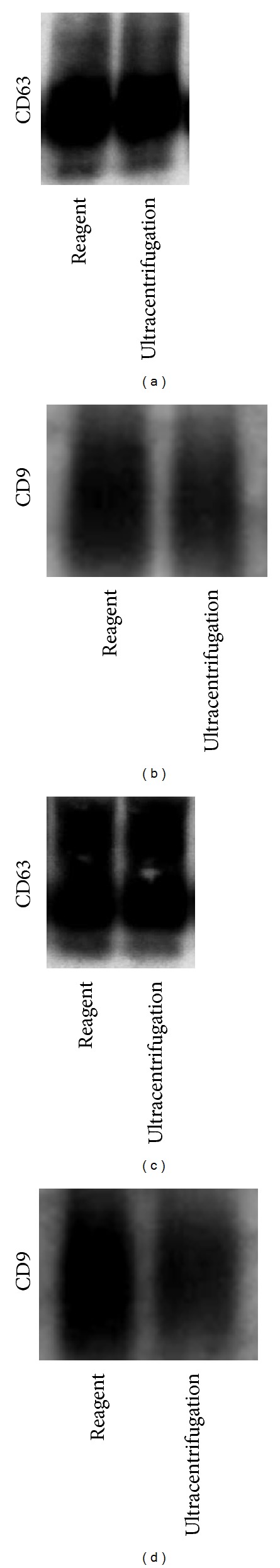
Western blot analysis for the presence of exosomal marker proteins CD63 and CD9 in HeLa cell culture media ((a), (b)) and serum ((c), (d)) derived samples. Exosomes isolated with either the Total exosome isolation reagents or ultracentrifugation protocols were separated on 4–20% Tris-Glycine gels. Standard Western blot procedures with anti-CD63 and anti-CD9 antibodies were used to detect exosomal protein markers.

Samples were next analyzed by Western blotting with antibodies specific to tetraspanins CD63 and CD9-well characterized exosomal markers [11, 31]. Results are shown in Figures 2(a) and 2(b) (cell media) and Figures 2(c) and 2(d) (serum), confirming that CD63- and CD9-positive nanovesicle populations were recovered with both reagent and ultracentrifugation. The above results demonstrate that both protocols isolate clean exosome populations, but the reagent consistently recovered more exosomes, and the protocol is much faster, easier, and more reliable.

### 3.2. Isolation of Exosomal RNA Cargo and Analysis by qRT-PCR

Once the purity of recovered exosomes was confirmed and the amount determined, we proceeded with the isolation and subsequent analysis of the exosomal RNA. Using the Total exosome RNA and protein isolation kit, developed specifically for this purpose, samples were put through an organic extraction followed by immobilization of RNA on glass-fiber filters, to purify the total RNA. We followed this protocol to isolate RNA from exosomes derived from HeLa cell culture media and blood serum samples using both the Total exosome isolation reagents and ultracentrifugation procedure. Subsequent analysis with Nanodrop and Bioanalyzer has shown that for exosomes isolated from 30 mL of HeLa cell culture media using the ultracentrifugation protocol ~3.8 ng RNA was recovered. The reagent method resulted in isolation of somewhat more exosomes from the same volume of HeLa cell culture media, from which ~7.5 ng RNA was extracted. For exosomes isolated from 4 mL of serum using the ultracentrifugation protocol ~0.9 pg RNA was recovered. The reagent method resulted in isolation of somewhat more exosomes from the same volume of serum, from which ~2 ng RNA was extracted.

**Figure 3:**
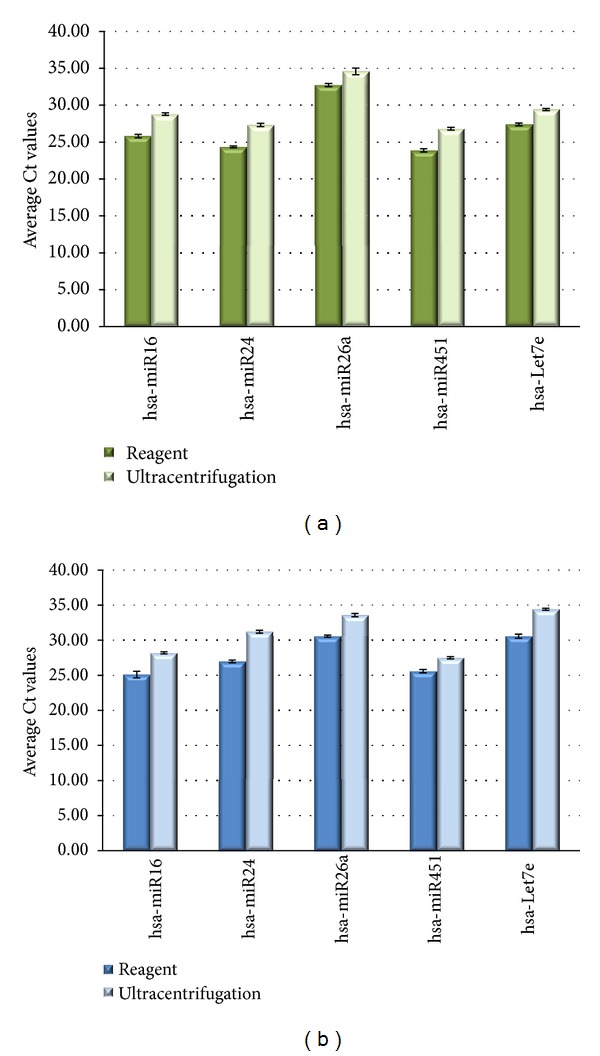
Analysis of the exosomal miRNA levels in HeLa cell culture media (a) and serum preparations (b) by quantitative RT-PCR. RNA was isolated using the Total exosome RNA and protein isolation kit from exosomes extracted using the Total exosome isolation reagents and the ultracentrifugation protocols. Levels of five microRNAs (miR16, miR24, miR26a, miR451, and let7e) were quantified by qRT-PCR using TaqMan assays and reagents.

Before proceeding with the sequencing analysis of the RNA, the levels of five microRNAs miR16, miR24, miR26a, miR451, and let7e, earlier reported to be present in exosomes [11, 21], were quantified by qRT-PCR. Results are displayed in Figure 3(a) (cell media) and Figure 3(b) (serum). Based on Ct values, 25–33 for all analytes, RNA isolation was efficient and the amount of material recovered is ample for standard PCR analysis—RNA recovered from exosomes derived from 3 *μ*L serum or 30 *μ*L cell media is sufficient for one qPCR reaction. The reagent method recovered somewhat higher levels to recover of exosomes compared to ultracentrifugation procedure, as indicated by 1–3 Ct shift for different RNAs. From this point, we focused on analysis of the RNA content of exosomes isolated with the reagent.

### 3.3. Preparation of the Small RNA Libraries and Sequencing Exosomal RNA

Exosomal RNA recovered from cell culture media and serum derived samples was analyzed by sequencing and compared to the parental samples, whole cells and cell-free serum, respectively. For the library construction, the primer binding flanking sequences were ligated to the RNA in the samples, followed by reverse transcription and PCR amplification. Certain modifications were introduced to the standard protocol to address the specific nature of the exosome samples: longer ligation and extended amplification were performed, as relatively low amount of RNA is recovered from the exosomes. Once the libraries were constructed, they were sequenced using the Ion Torrent PGM instrument, with 318 chips, following standard protocols.

### 3.4. Mapping and Counting RNA Sequencing Reads

The PGM 318 chips hold over 11 million wells. For the samples derived from cell media as well as serum, 9-10 million wells were loaded (75–90%) and sequenced. After subtracting the polyclonals, low quality sequences, and nontemplated ISPs, 5-6 million final readouts were obtained from each run. Of those reads, 90–98% were mapped. 

**Figure 4:**
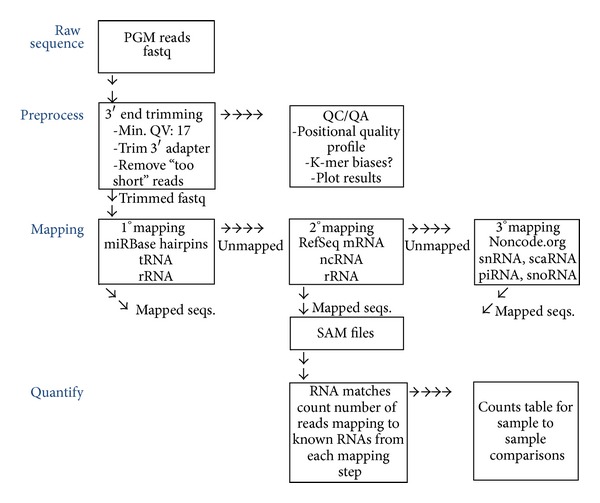
Iterative mapping pipeline used to map and quantify RNAs sequenced from both exosomes and parental samples. Each read that aligns to a particular RNA is referred to as a “count” of that RNA type. Quantitation is accomplished by calculating a tally of the counts for each type of RNA.

An iterative mapping pipeline was used to map and quantify RNAs sequenced from both exosome and parental samples (Figure 4). Each read, which aligns to a particular RNA, is referred to as a “count” for that RNA type. Quantitation was accomplished by calculating a tally of counts for each sample and RNA type.

Since no *a priori *exosome RNA content information is known for HeLa cell culture media, no assumptions were made about what databases should be referenced for mapping, quantitation, and comparison. For this reason, we designed an iterative mapping strategy for both sample types that included three mapping references utilized in a specific order. This is a “catch all” approach that takes advantage of published transcriptome annotations and sequences which are publicly available. The implementation of this approach involves a very intuitive workflow. Briefly, reads were first mapped to miRBase [41] microRNA precursors (hairpins). Any unmapped reads from this step were then mapped to NCBI RefSeq [42] transcripts. Finally, any remaining unmapped reads at this point were mapped to the Noncode.org noncoding RNA transcripts which include piwi-interacting (piRNA), small Cajal body-specific (scaRNA), and small nucleolar RNAs (snoRNA). At each step, mapped reads were counted per transcript and printed to a final report. The counts of mapped reads per transcript serve as proxies for representation and can be utlized for relative quantitative comparisons between samples. Results from the sequencing analysis will be discussed in the following sections looking at the specific RNA content from exosomes of each sample type.

### 3.5. Analysis of the Sequencing Data

#### 3.5.1. RNA Content of HeLa Cell Culture Media Derived Exosomes

**Figure 5:**
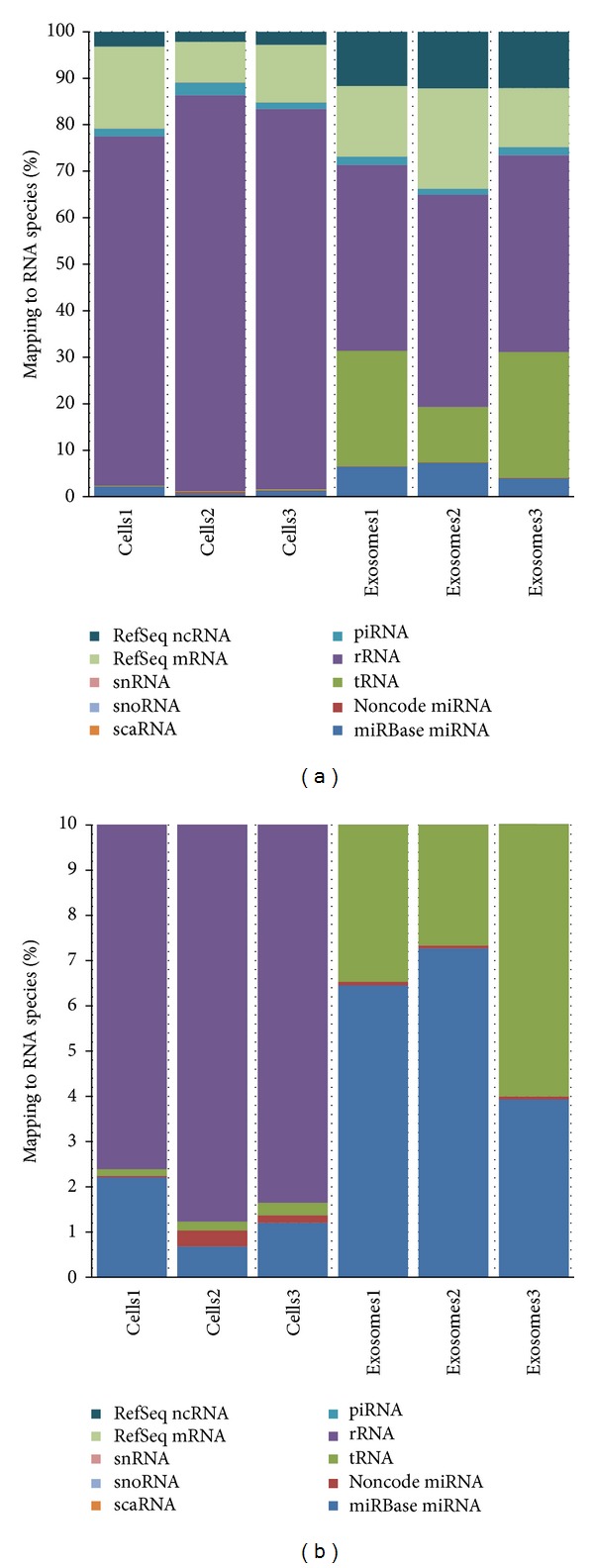
RNA sequencing results for exosomes versus parental HeLa cells. Stacked bar plots depict mapped read counts distributed by RNA species for both HeLa cells and HeLa cell culture media derived exosomes preparations. (a) Full scale. (b) Zooming into 0–10% range on the *y*-axis to view less prominent RNA types representation.

Using mapped sequence read counts, we quantified the RNA cargo of exosomes extracted from HeLa cell culture media and then compared it to RNA from the “parental” cells. This is, to our knowledge, the first report on RNA content of exosomes secreted by HeLa cells and also comparing exosomes to the parental samples. As illustrated in Figure 5, exosomes derived from HeLa cell culture media contained extremely diverse RNA “cargo.” As expected from previous research, a large number of miRNAs and mRNAs were detected. However, significant amounts of rRNA, tRNA, and other RNA types were discovered as well. There were clear differences in RNA species representation between cells and exosome preparations. The cell preparation had a high rRNA representation, about twice the amount in exosomes (~40%). piRNA was present in comparable amounts in both sample types, while miRNA, mRNA, RefSeq ncRNA, and tRNA representation in the exosomal samples was higher than that of parental cell preparations. This observation agrees with the aforementioned studies in which full length RNA was found to be represented in exosomes and may have a role in intercellular communication. 

When summarizing total counts per samples type, we calculated mean raw counts per RNA-Seq library and then ranked them from high to low for miRNA, tRNA, mRNA, and ncRNA categories. The top ten are listed in Table 1. In this view, representation is not given as a normalized percentage but as ranks of individual transcripts by category and may be compared directly between exosomal and parental cell samples. For exosomes, the transcripts that stand out include hsa-mir-21, hsa-mir-3160-1, chr6.trna61-MetCAT, chr1.trna109-GluCTC, SUSD2 mRNA, RNY5 ncRNA, and RNY4 ncRNA. The biggest overlap between exosomes and parental sample was observed for tRNA: 7 out of 10 top represented sequences were the same; for miRNA, 4 of 10 sequences were the same; for mRNA and ncRNA, 2 sequences were the same.

**Figure 6:**
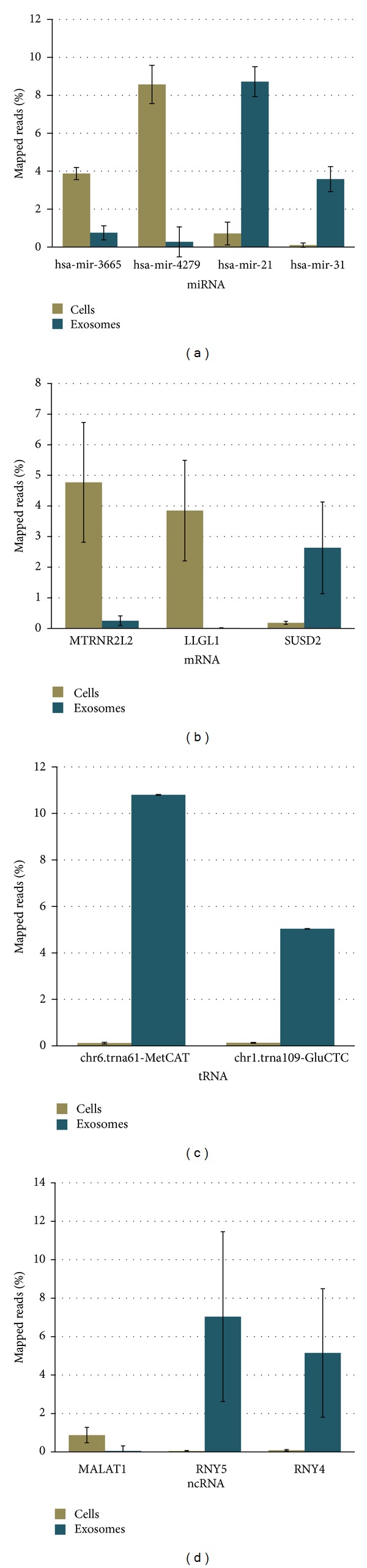
Differential representation of specific RNA in exosomes versus parental HeLa cells. miRNAs (a), mRNA (b), tRNA (c), and ncRNA (d) from cells and exosome preparations. Quantities were normalized by the percentage of reads aligned to that transcript out of the total mapped.

Several targets from four of the different HeLa major RNA species (miRNA, tRNA, mRNA, and ncRNA) showed significant differences in representation between the two sample types (Figure 6). In particular, miR-21, miR-31, SUSD2 mRNA, tRNA61-MetCAT, tRNA109-GluCTC, RNY5 ncRNA, and RNY4 ncRNAs were higher in exosomes, while miR-3665, miR-4279, MTRNR2L2 mRNA, LLGL1 mRNA and, MALAT1 ncRNA were significantly higher in parental cell samples. The reason for these differences is yet to be explored and will provide useful information regarding sorting of particular RNA sequences into exosomes. Interestingly, miR-21 has been reported to be a prognostic indicator in several types of cancer [43–45], and both miR-31 and miR-21 have been reported to be associated with esophageal cancer [46]. This raises the possibility that a mechanism by which metastases are induced within a cell population could be via intercellular communication by exosomes containing specific miRNAs.

It is also worth noting from Figure 6 the level of variability (error bars) seen within replicate libraries prepared from three separate HeLa cell cultures. Higher count variability was observed for mRNA and ncRNA measurements when compared to miRNA or tRNA measurements. This suggests that our libraries contain varying levels of natural degradation products and representation of mRNA and ncRNAs from exosomes are stochastic in nature. Moreover, the lower variability in miRNA representation could be an inference to functional importance. 

#### 3.5.2. RNA Content of Human Blood Serum Exosomes

**Figure 7:**
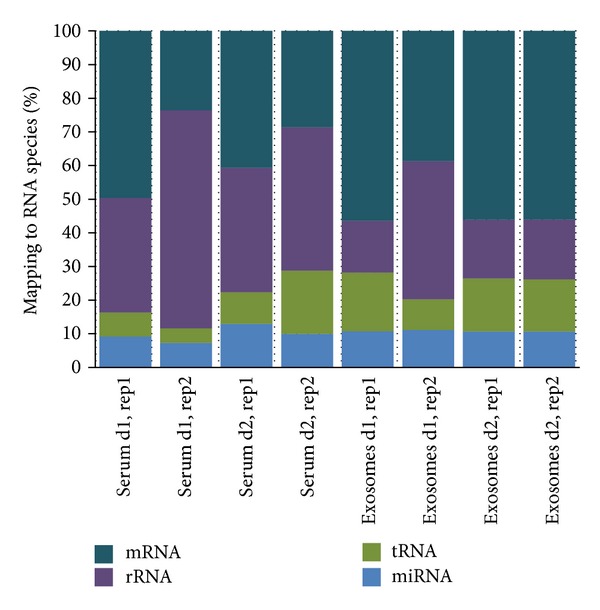
RNA sequencing results for exosomes versus parental serum. Stacked bar plot depicts mapped read count distribution by RNA species derived from two human blood donors, d1 and d2. Two replicates (rep1, rep2) were sequenced per donor. SnRNA, ScaRNA, SnoRNA, piRNA were also detected though at the very low levels.

Cell culture is a useful model for initial characterization studies; however, blood samples are of higher interest due to their clinical relevance and potential for use in human diagnostics. In the latter case more heterogeneity in RNA composition should be obviously expected versus cell culture since the samples are taken from multiple individuals. We analyzed the RNA cargo of exosomes extracted from the blood serum of healthy human donors, as well as “parental” serum samples. As illustrated in Figure 7, exosomes derived from serum contain diverse RNA “cargo”: miRNA, mRNA, rRNA, tRNA, and various ncRNA. There were clear differences in RNA species representation between whole serum and exosome preparations. Serum preparation had a higher rRNA representation, whereas mRNA, tRNA, and ncRNA representation in the exosomes was higher than that in serum preparations. miRNA was present in comparable amounts. However, when scrutinizing individual miRNA representation as a fraction of total mapped reads, we did observe a set of miRNAs which are differentially represented between serum and serum derived exosome fractions (as discussed later). 

When RNA composition profiles were compared between serum and cell culture samples, general differences were observable. Notably, rRNA read counts compose a smaller percentage of total reads in serum while levels of mRNA/ncRNA make up a higher percentage irrespective of sample replicate or type. In addition, the serum and exosomal libraries showed more variability in terms of RNA species distribution in duplicate libraries made from two blood donors. We expect that donor to donor variability may be a challenge in serum samples moving forward, so there is a need to further explore methods to reduce or account for this variability for future studies. 

Table 2 shows the representation of top 10 targets from each RNA species present in serum: miRNA, tRNA, mRNA, and ncRNA. For exosomes, the sequences that stand out include hsa-mir-1246, hsa-mir-451, chr6.trna4-ArgTCG, chr6.trna31-SerGCT, CCDC96 mRNA, HGSNAT mRNA, RNY4 ncRNA, and RNY5 ncRNA. The highest overlap between exosomes and parental serum was observed for tRNA and mRNA: 7 out of 10 top represented sequences were the same in both cases; for miRNA, 6 sequences out of 10 were the same; for ncRNA, 4 sequences were the same. Except for ncRNA, there was little overlap between most represented sequences in serum derived exosomes and HeLa cell derived exosomes.

**Figure 8:**
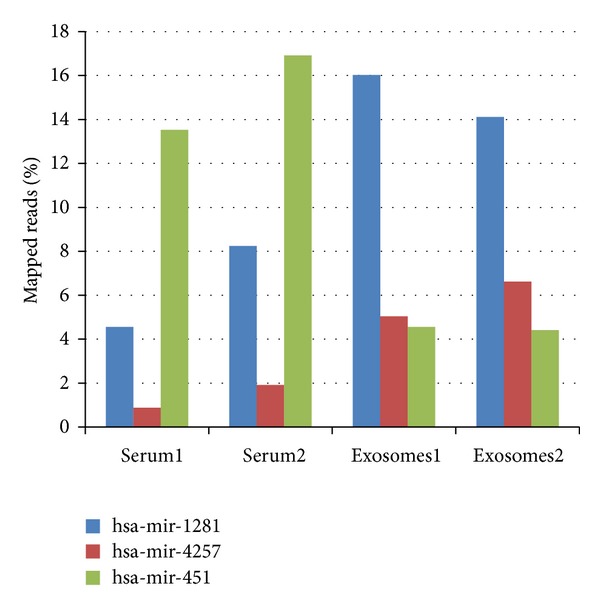
Differential representation of specific miRNAs in exosomes versus parental serum samples. Quantities were normalized by the percentage of reads aligned to that transcript out of the total mapped.

Levels of certain RNA sequences were substantially different between exosomes and parental serum sample as illustrated in Figure 8. A subset of 3 miRNA targets showed different representation profiles between the two sample types: miR-1281 and miR-4257 were higher in exosomes, while miR-451 was significantly higher in parental sample. miR-451, for instance, was reported as associated with resistance to chemotherapy in ovarian cancer [47] as well as playing a part in controlling glioma cell proliferation and migration [48]. In additional, it has also been reported as potential marker for renal cell carcinoma with much lower levels in RCC patients [49]. This miRNA has a number of important intracellular functions, but potenially no value for cell signaling purposes, and thus its levels within exosomes are low. The above miRNAs could potentially be utilized as exosomal markers, as currently available options are limited to a handful of proteins including CD63, CD81, and CD9, none of which seems to be absolutely specific to exosomes. 

The mechanism of sorting particular RNA sequences into exosomes is not well understood at the moment, and is extremely challenging to study. Each cell secretes several types of exosomes and microvesicles, with different functions and containing different cargo. For example some of these vesicles simply allow cells to expel unnecessary RNA and proteins, while others allow cells to send signaling or affector molecules to other cell populations within the body [6]. When analyzing exosomes from body fluids, the situation becomes increasingly complicated, as many cell types contribute exosomes to the population. Vitally needed is the development of tools allowing the study of the mechanisms of formation of the exosomes, their secretion, trafficking, and internalization in the recipient cells. It is also crucial to enable separation of various classes or subtypes of the exosomes, based on their surface protein signature or other parameters.

## 4. Conclusions

We have successfully developed a set of reagents and a complete exosome workflow solution, starting from fast and efficient extraction of exosomes from cell culture media and blood serum to robust isolation of RNA and characterization of exosomal RNA content using qRT-PCR and next-generation sequencing methods. The procedure is completed in a fraction of the time, compared to the current standard protocols utilizing ultracentrifugation, and allows recovery of fully intact exosomes in higher yields. Analysis of the sequencing data revealed a very sophisticated RNA content in exosomes, with most of the cellular coding and noncoding RNA present in exosomes but in many cases at significantly different levels and ratios. It will be of paramount importance to gain a better understanding regarding the sorting of particular RNA sequences into exosomes and further into different exosome subpopulations. The work described here is the first step towards developing standardized techniques and protocols for isolation of exosomes and downstream analysis of their constituents. These reagents and workflows will be highly useful for scientists working on both basic research aspects of exosomes and developing next-generation minimally invasive diagnostics.

## Figures and Tables

**Table 1a:** (a)

miRBase ID	Cells	miRBase ID	Exosomes
hsa-mir-4279	731.00	hsa-mir-21	2015.33
hsa-mir-1234	505.00	hsa-mir-3160-1	1007.33
hsa-mir-451a	410.33	hsa-mir-4739	894.33
hsa-mir-3665	330.33	hsa-mir-31	827.33
hsa-mir-4449	265.67	hsa-mir-23a	783.67
hsa-mir-3960	240.67	hsa-mir-24-2	644.00
hsa-mir-3160-1	238.00	hsa-mir-1273a	521.67
hsa-mir-1273a	233.67	hsa-mir-30a	439.33
hsa-mir-1273g	195.33	hsa-mir-451a	371.33
hsa-mir-92b	180.00	hsa-mir-1273g	368.00

tRNA ID	Cells	tRNA ID	Exosomes

chr1.trna109-GluCTC	175.33	chr6.trna61-MetCAT	34129.67
chr16.trna34-GlyCCC	147.33	chr1.trna109-GluCTC	15924.33
chr16.trna18-GlyGCC	121.67	chr16.trna18-GlyGCC	5696.67
chr6.trna61-MetCAT	101.00	chr1.trna33-GlyGCC	2354.33
chr17.trna28-CysGCA	86.33	chr16.trna10-LysCTT	2119.67
chr5.trna5-ValAAC	52.67	chr5.trna11-LysCTT	2107.33
chr5.trna11-LysCTT	48.00	chr13.trna3-GluTTC	1987.00
chr1.trna33-GlyGCC	35.67	chr16.trna34-GlyCCC	1928.33
chr17.trna10-GlyTCC	33.00	chr1.trna54-GluCTC	1450.33
chr13.trna3-GluTTC	25.33	chr1.trna124-GlyCCC	1365.00

**Table 1b:** (b)

mRNA	Accession	Cells	mRNA	Accession	Exosomes
MTRNR2L2	NM_001190470	4793.67	SUSD2	NM_019601	3172.33
MTRNR2L8	NM_001190702	2646.33	BRWD3	NM_153252	501.00
SETD2	NM_014159	544.67	SENP6	NM_015571	362.67
CUX1	NM_001202543	475.33	FAM59B	NM_001191033	355.33
TUBA1C	NM_032704	475.33	TUBBA4	NM_006087	338.33
FXR1	NM_004860	363.33	QRFPR	NM_198179	313.33
SYT6	NM_001253772	319.00	MDK	NM_001012334	307.00
FUS	NM_001170634	287.33	MTRNR2L2	NM_001190470	302.00
MDK	NM_001012334	238.67	CWC25	NM_017748	266.00
UBE2S	NM_014501	230.33	DUSP13	NM_001007272	232.00

ncRNA	Accession	Cells	ncRNA	Accession	Exosomes

SNORA72	NR_002581	3865.67	RNY5	NR_001571	8482.67
MALAT1	NR_002819	879.67	RNY4	NR_004393	6206.33
RNU1-9	NR_004426	798.00	RN5S3	NR_023365	2343.67
RN5S1	NR_023363	668.67	RNY1	NR_004391	2109.67
RNU2-1	NR_002716	629.00	RNY4P8	NR_033356	2094.33
RN7SL1	NR_002715	581.67	RNY3	NR_004392	1119.33
SNORD3C	NR_006881	531.00	RN7SL2	NR_027260	702.33
VDAC1	NR_036624	437.33	SNORD31	NR_002560	619.33
RN7SK	NR_001445	435.33	RNU1-5	NR_004400	561.67
RN7SL2	NR_027260	425.67	RN7SL1	NR_002715	457.33

**Table 2a:** (a)

miRBase ID	Serum	miRBase ID	Exosomes
hsa-mir-1246	5222.5	hsa-mir-1246	2145.0
hsa-mir-451	2163.5	hsa-mir-451	1837.0
hsa-mir-2115	77.0	hsa-mir-3934	1444.5
hsa-mir-1281	531.0	hsa-mir-1281	1068.5
hsa-mir-486	337.5	hsa-mir-3180-1	871.0
hsa-mir-16-1	326.0	hsa-mir-4257	851.5
hsa-mir-3180-1	116.5	hsa-mir-181d	687.5
hsa-mir-4257	525.0	hsa-mir-486	593.5
hsa-mir-124-1	136.0	hsa-mir-766	580.5
hsa-mir-3167	293.5	hsa-mir-497	565.0

tRNA ID	Serum	tRNA ID	Exosomes

chr6.trna31-SerGCT	801.0	chr6.trna4-ArgTCG	2071.0
chr12.trna10-AspGTC	718.0	chr6.trna31-SerGCT	1867.0
chr6.trna4-ArgTCG	661.5	chr15.trna4-ArgTCG	1010.0
chr15.trna4-ArgTCG	475.0	chr6.trna14-TyrGTA	894.5
chr14.trna19-TyrGTA	364.5	chr14.trna19-TyrGTA	884.5
chr6.trna124-ArgTCG	237.5	chr7.trna7-CysGCA	681.5
chr6.trna14-TyrGTA	223.5	chr1.trna105-GlnCTG	591.0
chr12.trna12-AspGTC	214.0	chr6.trna52-ArgTCT	581.0
chr6.trna145-SerAGA	211.0	chr6.trna124-ArgTCG	565.5
chr6.trna52-ArgTCT	200.5	chr16.trna1-ArgCCG	544.5

**Table 2b:** (b)

mRNA	Accession	Serum	mRNA	Accession	Exosomes
CCDC96	NM_153376	251.5	CCDC96	NM_153376	606.5
SIRPG	NM_080816	240.5	HGSNAT	NM_152419	503.0
TBX22	NM_016954	215.0	TBX22	NM_016954	465.0
RNF223	NM_001205252	200.5	SIRPG	NM_018556	350.0
SIRPG	NM_018556	198.0	SIRPG	NM_080816	340.0
NFIX	NM_002501	198.0	NFIX	NM_002501	338.5
RAP2A	NM_021033	174.0	GPR126	NM_198569	288.0
SIRPG	NM_001039508	154.0	ILDR2	NM_199351	273.5
HGSNAT	NM_152419	143.0	PAPOLB	NM_020144	267.5
RBFOX1	NM_001142333	129.5	SIRPG	NM_001039508	246.5

ncRNA	Accession	Serum	ncRNA	Accession	Exosomes

RNY4	NR_004393	1043.5	RNY4	NR_004393	704.5
RNY5	NR_001571	477.0	RNY5	NR_001571	262.0
RNY3	NR_004392	360.0	RNY3	NR_004392	174.0
RNY4P8	NR_033356	177.0	FLJ33534	NR_040080	167.0
RNU2-1	NR_002716	159.5	LOC100130673	NR_038454	155.0
RNY1	NR_004391	145.5	LOC100507605	NR_038375	153.0
RNU12	NR_029422	127.5	C20orf173	NR_026933	152.0
C1orf213	NR_033690	101.0	LOC100507605	NR_038374	139.5
RNU5B-1	NR_002757	71.0	LOC644936	NR_004845	136.0
C20orf173	NR_026933	70.5	LOC100507605	NR_038376	130.5
